# Delays in the vaccination of infants between 2 and 18 months of age: associated factors in Chile

**DOI:** 10.1186/s12889-023-16769-3

**Published:** 2023-09-28

**Authors:** Paula Leal, Jorge Gaete, Cecilia González, Pamela Burgos

**Affiliations:** 1 Faculty of Medicine, Departamento de Epidemiología y Estudios en Salud, Universidad de los Andes, Santiago, Chile; 2 Research Center for Students Mental Health (ISME), Faculty of Education, Universidad de los Andes, Santiago, Chile; 3 Millennium Science Initiative Program, Millennium Nucleus to Improve, the Mental Health of Adolescents and Youths, Imhay, Santiago, Chile; 4 Immunization Department, Ministry of Health, Santiago, Chile

**Keywords:** Delays, DTaP, Vaccination, Hesitancy, Questionnaire, Trust and positive attitudes

## Abstract

**Introduction:**

Infant vaccination has significantly reduced the morbidity and mortality of transmittable diseases worldwide. Its coverage is high (85%); however, partial or suboptimal vaccination has been an important public health problem. This study aimed (1) to design and explore the psychometric features of a questionnaire to determine the reasons for this partial or suboptimal vaccination; and 2) to determine the factors associated with delaying Diphtheria, Tetanus, Poliomyelitis (DTaP) vaccination.

**Material and methods:**

This study contained two parts. In Part One, a questionnaire was created by the research team and then validated by a committee of experts in the field and a group of parents. It included the following contents: sociodemographic variables, features of the vaccination services, history of vaccination, and attitudes and perceptions about vaccination. Part Two was a cross-sectional study, recruiting private and public healthcare centers to explore the psychometrics features of the instrument, performing exploratory factor analysis, and determining the associated factors with DTaP vaccination delay throughout multivariable regression models.

**Results:**

Initially, six experts validated the questionnaire. For instance, on a scale of 1 to 5, the general evaluation of the questionnaire was ≥ 4 for all the experts. Additionally, five experts considered that most of the questions were easy to understand, and all thought the questionnaire had a clear and logical organization. The resulting questionnaire included the “Trust and positive attitude towards vaccination” scale, which had a good structure of items and internal consistency (α = 0.7918). Six healthcare centers were recruited in the second part of the study, and 715 people answered the questionnaire. Not being the mother who brings the child to the health center, having more than one child, and having a history of previous vaccination delays increased the risk of delaying vaccination. Attending the healthcare center for a reason other than only vaccination, obtaining information about vaccines from the Internet, and having higher trust and positive attitudes to vaccination reduced the risk of delay.

**Conclusions:**

First study during the pandemic to explore the role of different factors on the risk of DTaP vaccination delay in Latin America. The findings highlighted the importance of trust in the vaccination system. The instrument presented in this article may help the scientific community evaluate future interventions to increase trust and positive attitudes toward the vaccination process.

**Supplementary Information:**

The online version contains supplementary material available at 10.1186/s12889-023-16769-3.

## Introduction

Vaccination has significantly reduced the morbidity and mortality of immune-preventable diseases worldwide. Prepandemic, the global rate of Diphtheria, Tetanus, Poliomyelitis (DTaP) vaccination coverage in children under two years old has remained stable at 85%, with no significant changes being recorded over the last years [1]. During the pandemic, the World Health Organization called all countries to keep their immunization program and ensure the best sanitary conditions to execute this plan [2].

A high level of coverage does not guarantee adequate infection protection for infants [3, 4], mainly because delays or having received fewer doses may be as high as 50% [5, 6]. This partial vaccination leaves infants susceptible to diseases and reduces collective immunity [7, 8]. For this reason, several international organizations have stressed the importance of timely vaccination as a core measure associated with high vaccine coverage in the population [9].

Recent studies have identified parental attitudes and opinions regarding vaccines as critical reasons for partial vaccination [10]. In this regard, Opel et al. [11] developed and validated the “Parent Attitudes about Childhood Vaccines (PACV)” tool as a first intent to explore parental attitudes, which comprises 15 items in 3 domains: behavior, safety, and effectiveness of the vaccine, and general attitudes about the vaccine [12]. This instrument has been widely used in the United States [13], and translated and psychometrically tested in Italy [14] and Malaysia [15]. Nevertheless, this scale does not sufficiently explore the issue of partial vaccination. In this context, in 2014, PAHO/WHO made available to Latin American countries a standardized methodology for evaluating Missing Opportunities of Vaccination (MOV) to shed light on the factors associated with partial vaccination and non-vaccination considering the perspective of healthcare systems and the attitude of providers and users. These efforts aimed to implement specific corrective interventions in vaccination sites or target the demand for such services [16].

Other attempts to understand the issue of partial vaccination have focused on reports by health center staff. For example, a small survey on vaccination was piloted in the WHO Strategic Advisory Group of Experts (SAGE) in 2014 and completed by national immunization administrators worldwide [17]. This instrument aimed to collect local information about vaccination-related issues and offer suggestions for countries to develop suitable strategies and policies to address parents´ concerns and preserve trust in the vaccination [18]. Within the context of the 2021–2030 Global Vaccine Action Plan [19] and the work carried out by SAGE on vaccine hesitancy, new questions were considered and tested for inclusion in the 2012, 2013, and 2014 UNICEF Joint Reporting Form [17]. However, this was mostly a qualitative survey, with no information gathered from parents.

In Chile, the coverage in the DTaP vaccination, defined by calendar among infants, reached nearly 90% in 2019, according to the Ministry of Health [20]. However, an analysis of the data revealed a delay in the DTaP vaccination of the target population [20], showing that the vaccination is completed weeks or months later than the dates noted in the individual vaccination schedule. The information collected made it possible to follow up the schedules individually, revealing that 95% of breastfed babies receive the first DTaP dose at most 51 days after the age of eligibility, which reflects a good implementation with a high level of timely coverage. Nevertheless, when analyzing the coverage of the third dose, a target of 95% is reached with a delay of 330 days after the age of eligibility [20]. During the pandemic, in 2020, the execution of the immunization program was reduced by 0.39% for DTaP 1 and 12.02% for DTaP 4, compared to the period 2015–2019 [21].

No studies have been published in Chile regarding potential factors associated with vaccination delays and refusal. No validated instruments are available in Chile to study the phenomenon of vaccination delays and how parental factors such as attitudes and opinions are associated with this delay. The aims of this study were: (1) to design and explore psychometric features of a questionnaire to determine reasons for this partial or suboptimal vaccination, and 2) to determine the factors associated with delaying DPT vaccination in private and public centers in Santiago.

### Material and methods

We carried out the study in two parts: Part One, aiming to design and create the instrument, and Part Two, aiming to validate the instrument, and then analyze the data and explore the potential factors related to vaccination delay.

### Part one

#### Design and construction of the questionnaire

The basis of our questionnaire was based on the MOV instrument by PAHO/WHO [16], and the tools created by SAGE [22] and the PACV survey [11]. We also added specific aspects regarding the tutor's reasons for not vaccinating their child on scheduled dates.

### General questionnaire

The first draft of the questionnaire incorporated the following variables in 5 domains:

1. Sociodemographic variables: tutor and infant date of birth, tutor and infant sex (1 = Woman; 2 = Man), municipality of residence of the tutor (1 = Low-income municipality; 2 = Middle-income municipality; 3 = High-income municipality), nationality (1 = Chilean; 2 = Foreigner) and country of origin of the tutor and the infant (1 = Chilean; 2 = Foreigner), relationship between the tutor and the infant (1 = Father; 2 = Mother; 3 = Other), tutor marital status (1 = Married; 2 = Single; 3 = Separated/Divorced), among others.
2. Variables related to the health center: the main reason for taking the infant to this center (1 = To vaccinate the infant; 2 = Another reason), location of the health center regarding tutor's residence (1 = Located in the same municipality; 2 = Located in a different municipality), reasons for coming to this health center no located in their municipality (1 = Quality of care; 2 = Closeness), transport used to travel to this health facility (1 = Public transport; 2 = Personal transport [e.g., car]), time spent traveling to the healthcare center today (1 =  < 30 min; 2 = 30 or more minutes) and questions regarding their satisfaction with the health center.
3. Variables related to knowledge about vaccination schedule: dose scheduled for that day (1 = 2 to 6 months; 2 = 18 months), vaccination card availability (1 = Yes; 2 = No), knowledge the date of the next vaccination (1 = Yes; 2 = No), reasons for ignoring the date of the next vaccination appointment (1 = I was not told; 2 = I did not understand or it was unclear; 3 = I do not remember; 4 = Other reasons), among others.
4. Variables related to vaccine usefulness, vaccination delays, and vaccine reticence: “I know what vaccines are used for in general” (1 = I know what they are used for; 2 = I do not know what they are used for), source of information about vaccines (1 = Health professionals; 2 = Family and Friends; 3 = Internet); “It has happened before that you have not been able to vaccinate your child (1 = Not been able to vaccinate the child, 2 = Yes, I was able to vaccine the child); “prior delays in vaccination” (1 = History of prior delays; 2 = No history of prior delays), “reasons for prior delays” (1 = Problems with the infant or tutor; 2 = Issues due to the healthcare centers' location, opening hours, accessibility, and/or distance; 3 = Fear of COVID-19), history of deciding not to vaccinate the infant (1 = Yes; 2 = No), reasons for not vaccinating the infant (1 = Fear of vaccine risks, 2 = Expensive complementary vaccines; 3 = Fear of COVID-19), among others.
5. Trust and Positive Attitudes Towards Vaccines Scale: The research team created an instrument with six items assessing the degree to which parents or caregivers agree with statements regarding trust in vaccination: 1. Vaccines are good and safe, 2. Vaccination protects my child from diseases, 3. In general, I do what physicians recommend me regarding the vaccines for my child, 4. The information posted in the vaccination calendar seems adequate and I trust it, 5. I am satisfied with all the vaccines that are available in the vaccination schedule, and 6. The information I receive about adverse events (or unwanted reactions) seems adequate to me and gives me confidence. The answers ranged from 1 (“Strongly disagree”) to 5 (“Strongly agree”). The total score was calculated as the mean score of all items.

The resulting instrument was reviewed by a group of six experts, distinguished in the field of vaccines in Chile: a journalist, a PAHO representative, a nurse, a pediatrician specialized in infectious diseases, a pediatrician specialized in immunology, and an infectious disease specialist. Each expert produced comments and rated the instrument through an online form from 1 (Very poor) to 7 (Outstanding), on the following topics: general evaluation of the survey (length and understanding), pertinence of the questions, and adequacy of the contents covered. The experts' responses and suggestions were analyzed and incorporated into the questionnaire.

After implementing the experts' suggestions, the modified questionnaire was piloted to a convenience sample of 15 participants (all mothers) who attended the vaccination center of a pediatric Hospital (Santiago) in November 2020 to receive the 2-, 4-, 6-, or 18-month DTaP vaccine doses. The objective of this evaluation was to determine the degree to which the respondents understood the questions, their understanding of the organization of the sections, and the length of the questionnaire. The rating score was 1 (Very poor) to 7 (Outstanding). See the final questionnaire in Suppl 2.

### Part two

#### Design

Cross-sectional study with convenience sampling procedure.

#### Participants

The sample was obtained using the records of the National Registry of Immunizations (Registro Nacional de Inmunizaciones, RNI) who had received the DTaP vaccine (aged 2, 4, 6, and 18 months) at six healthcare centers of several municipalities of the Metropolitan Region. The healthcare centers have a vaccination center in the same building as an integral part of the health center and were selected based on an analysis of the number of vaccines administered and the delay levels detected in the administration of the DTaP vaccines in 2019, according to the nominal electronic registry in the RNI across all the healthcare centers of the Metropolitan Region. The healthcare centers were ranked according to the number of delays logged in their records. Based on this information, we selected the 3 private and 3 public healthcare centers with the largest number of delays.

### Sample size

To determine the number of subjects who would participate in the study, we estimated a sufficiently large sample size that would enable us to evaluate the psychometric properties of the instrument. Even though there is no clear or consensual recommendation of the sample size needed to conduct a factor analysis, several authors have recemented to include at least 300 subjects [23] or ten subjects per item [24, 25], whichever is larger. Since the questionnaire comprised 41 questions, 410 subjects were expected to participate. It was also important to have an equivalent number of on-schedule and delayed participants as well as private and public healthcare centers (both being stratification variables). It is also worth noting that epidemiological studies of this type generally have a level of rejection from participants of about 50% [24]. Given these conditions, the expected sample size was 616 participants, with 308 per type of health center (private and public) and 308 per vaccination status (on schedule and delayed). This was done to enable us to compare populations according to the functioning of the instrument and associated variables.

### Recruitment procedure

First, we contacted the Directors of the selected healthcare centers. The state centers were the Family Health Center (Centro de Salud Familiar, CESFAM) Cruz Melo, CESFAM Domeyko, CESFAM No. 1 Ramón Corbalán Melgarejo; and the private health centers were Clínica Dávila, Clínica Indisa, and Clínica Santa María. After their approval, we initiated the administration of the survey in person in each health facility in December 2020 from Monday to Friday and from 9 am to 5 pm. Due to the COVID-19 pandemic affecting the country, we followed all the preventive protocols established by each healthcare center. The surveys were administered by a team of trained survey takers, who participated in a 2-h training program. To recruit interviewees at each healthcare center, we asked the center staff working at the vaccination unit to point out to the survey taker if the infant was on schedule or delayed with his/her vaccinations DTaP. All people attending the health center in December for vaccination of the 2-, 4-, 6-, or 18-month DTaP vaccine doses were eligible to participate in the study. Afterward, the center staff administered the DTaP vaccine (In Chile, the hexavalent vaccine). Then, the survey taker explained to the tutor the study's aims and the survey, gave and read out the informed consent, and asked the tutor to state whether he/she agreed or refused to participate. After signing the informed consent, the tutor completed the survey on a tablet computer. We used Google Forms to register the answers, and then the data was downloaded into Excel to then converted into a Stata data set.

### Statistical analysis

#### General results

Firstly, we described the sociodemographic variables and the items' psychometric characteristics by using descriptive statistics, including frequencies and percentages, and means and standard deviations when necessary.

### Dimensionality and reliability analyses of the “Scale of Trust and Positive Attitudes Towards Vaccines”

First, we calculated the frequency of responses for each item to explore the floor and ceiling effect. Then, we conducted an exploratory factor analysis (EFA). According to the nature of the data, we used polychoric correlations [26]. For factor extraction, we used principal axis factoring, recommended when multivariate normality is not completely fulfilled [27], with the promax oblique rotation method, which allows the factors to correlate [28]. We used parallel analysis [29] to identify the number of factors to include in the solution. This identification was performed by replacing the raw data method with an optimal implementation based on minimum rank factor analysis [30], generating 500 random correlation matrices. With this analysis, a factor is significant if the associated eigenvalue is larger than that corresponding to a given percentile, such as the 95th percentile of the distribution of eigenvalues derived from a random dataset. This method is the best available solution to decide the number of factors to retain for a given scale [31].

The reference values for the analysis of factor loadings are the following: factor loadings > 0.70 were deemed optimal, 0.40—0.70 moderate, and 0.30—0.39 minimal [32]. Reliability was assessed using Cronbach’s alpha coefficient using the following parameters: alpha ≥ 0.9 is “Excellent”; 0.9 > alpha ≥ 0.8, “Good”; 0.8 > alpha ≥ 0.7, “Acceptable”; 0.7 > alpha ≥ 0.6, Questionable; 0.6 > alpha ≥ 0.5, “Poor,” and 0.5 > alpha, “Not acceptable” [33, 34]. The final score was calculated using the average score for each item with a valid response.

### Association analyses

We defined “Delay in vaccination” as the main dependent variable. We gathered information about how many days had passed between the scheduled date for vaccination and the current day of vaccination. If this period was over 28 days, it was regarded as a delay in receiving the scheduled vaccine [16]. In the association analyses, we included all the independent variables for the five domains presented in the questionnaire. For the purposes of the analyses, when possible, the independent variables were categorized as dichotomous variables to simplify the interpretation of the results. For the full list of response options for each variable, see Suppl 2. When examining highly related variables (e.g., Nationality and Country of origin), we used only one of them. Initially, we conducted univariate logistic regression analysis for each of the independent variables (Model 0); second, we explored the association of all significant independent variables with “Delay in vaccination” in Model 0 (using a cut-off point of p < 0.2), performing later a multivariate logistic regression analysis (Model 1) within each domain as presented in the General questionnaire section above. Finally, the association between “Trust and positive attitudes towards vaccines” and “Delay in vaccination” was explored, adjusted for the sociodemographic variables that were significantly associated with “Delay in vaccination” (Table 8 and Table 12). The present analysis is considered exploratory to identify potentially modifiable variables, which may be examined fully in a larger epidemiological study or in future interventions.

The cut-off point for statistical significance was established at *p* < 0.05, and the confidence intervals were reported. All analyses were performed in STATA 14.0. All analyses were conducted using the full dataset.

## Results

### Part one

#### Design and construction of the questionnaire

##### Experts’ assessments

The questionnaire, in general, was rated 6 points by 3 experts, 5 points, by one of them, and 4 points by 2 of them in a score from 1 to 7. With respect to the pertinence of questions in the instrument, it was rated 7 points by 50% (*n* = 3) of the respondents, 6 points by 2 of them, and 5 points by one of them. The majority of experts (5/6) considered that most of the questions were easy to understand, and all of them considered that the questionnaire had a clear and logical organization.

The experts made suggestions regarding 61.8% of the questions (21/34 items), 19 of which were implemented. The suggestions mostly consisted of changes to the wording of some items to make them easier to understand. The suggestions that were not accepted concerned two items: i) the question on marital status (one expert asked that it be removed because he deemed it unnecessary; however, the research team decided to keep it in the questionnaire because it was an important demographic question), and ii) the question on educational level (one expert requested that the term “preparatoria” be removed because although this category refers to a primary educational level, it is not commonly used in Chile, where the term “básica” is preferred; nevertheless, the research team decided to keep both terms in view of the high rates of migration from other Latin American countries where the term “preparatoria” is used).

Furthermore, the experts suggested adding 12 questions, 9 of which were accepted by the research team and prepared for inclusion in several sections of the instrument. For instance, sociodemographic data were complemented with time of residence (in years), type of health insurance of the tutor and infant (1 = Public; 2 = Private), and family structure (1 = Single parent; 2 = Biparental and/or extended). Regarding the participants' vaccination history and opinions, the following questions were incorporated: “Has been unable to vaccinate the infant in the past”, and if this “has happened more than once” (1 = Yes; 2 = No). Also, we added questions on vaccination delays and vaccination reticence, such as sources of health information accessed (1 = Health professional; 2 = Family and friends; 3 = Internet), whether the respondent changed his/her opinion regarding vaccination rejection (1 = Yes; 2 = No), and one open-ended questions asking why the respondent changed his/her mind about vaccination rejection. Due to the small number of participants who answered this question, we did not conduct any further analysis of these responses. The rest of the questions were related to the date of vaccination, attitudes towards vaccination, and perception of the general management of the health center. These questions were not included because these themes were already included in other questionnaire sections.

In a pilot study of the questionnaire with 15 participants, 93.3% (*n* = 14) of the respondents rated 7 points to the survey in general (scale ranged from 1 to 7), and 100% (*n* = 15) found all the questions in every section to be adequately understandable.

### Part two

#### Population

All eligible participants accepted to take part in the study and completed the questionnaire. We recruited 715 people from six health centers over approximately four weeks. See Suppl. Table 1. During this period, significantly fewer people attended the health center due to the pandemic and because all public health centers had suspended their activities due to a national strike ─due to health personnel demands for improved working conditions during the pandemic.

The number of respondents from the public (*n* = 333, 46.6%) and private health centers (*n* = 382, 53.4%) was similar. Most tutors were women (82.7%, *n* = 591), and the mother (82%, *n* = 586) of the infant. A 48,5% (*n* = 347) of infants were female. Most tutors were single (68.6%, *n* = 491), but had a biparental family (87.6%, *n* = 626). A 43.4% (*n* = 310) of participants lived in high-income municipalities, 31.5% (*n* = 225) in middle-income municipalities, and 25.2% (*n* = 180) in low-income municipalities. Most of the tutors (54%, *n* = 386) and infants (98%, *n* = 704) were Chilean. And 56.6% (*n* = 405) had public health insurance. See Table 1 and Suppl 1 Table 2, 3.

**Table 1:** Sociodemographic characteristics of the infant and the tutor

Variables	n	%	(95% CI)
**Sex of the tutor**			
Woman	591	82.7	(79.7–85.3)
Man	124	17.3	(14.7–20.3)
**Relationship between the tutor and the infant**			
Mother	586	82	(79.9–84.6)
Father	114	15.9	(13.4–18.8)
Other	15	2.1	(1.2–3.3)
**Marital status**			
Married	217	30.3	(27.1–33-8)
Single	491	68.7	(65.2–72.0)
Separated/Divorced	7	1.0	(0.4–0.2)
**Family structure**			
Biparental	626	87.5	(84.9–89.8)
Single parent	74	10.3	(8.3–12.8)
Extended	15	2.1	(1.3–3.4)
**Municipality of residence of the tutor**			
High-income municipality	310	43.4	(39.8–47.0)
Middle-income municipality	225	31.5	(28.2–35.0)
Low-income municipality	180	25.2	(22.1–28.5)
**Nationality of tutor**			
Chilean	386	54.0	(50.3–57.6)
Foreigner	329	46.0	(42.4–49.7)
**Nationality of infant**			
Chilean	704	98.5	(97.2–99.1)
Foreigner	11	1.5	(0.8–2.7)
**Type of health insurance of the tutor and infant**			
Public	405	56.6	(53.0–60.2)
Private	310	43.4	(39.8–47.0)
**Number of children of the mother**			
One child	459	64.2	(60.6–67.6)
More than one child	256	35.8	(32.4–39.4)
**Educational level of the tutor**			
With no higher education	211	29.6	(26.3–33.0)
With higher education, unfinished	86	12.1	(9.8–14.7)
With higher education, finished	386	54.1	(50.4–57.8)
With postgraduate studies	30	4.2	(2.9–5.9)
**Labor condition of the tutor**			
Unemployed	130	18.2	(15.5–21.2)
Paid work	584	81.8	(78.8–84.4)

### Variables related to the health center

Vaccinating the infant was the main reason for attending the health center (94.1%, *n* = 673). Most tutors resided in a municipality other than that where the health center was located, and the main reason for this was that they preferred the service provided there because of its quality. Most tutors used their own transport and required less than 30 min to get there. For more details, see Table 2 and Suppl 1 Table 4.

**Table 2:** Characteristics related to the health center

Variables	n	%	(95% CI)
**Main reason for bringing the infant to this center**			
To vaccinate the infant	673	94.1	(92.1–95.6)
Another reason	42	5.9	(4.4–7.8)
**The health center location regarding residence**			
Located in the same municipality of residence	331	46.3	(42.6–50.0)
Located out of the municipality of residence	384	53.7	(50.0–57.3)
***For those who go to the health center not located in municipality of residence, reasons:***			
Quality of care	215	56.0	(51.0–60.9)
Health insurance	69	18.0	(14.4–22.1)
Closeness	54	14.0	(10.9–17.9)
Use of health center for other health issues	46	12.0	(9.1–15.6)
**Means of transportation to travel to the health center**			
Public transport	134	18.7	(16.0–21.8)
Personal transport	581	81.3	(78.2–83.9)
**Time spent traveling to the healthcare center today**			
< 30 min	484	67.7	(64.2–71.0)
30 or more minutes	231	32.3	(29.0–35.8)
**Rating of several aspects of the health center (1 to 7)**	**Media**	**SD**	
Location	6.3	1.0	
Accessibility and distance	6.2	1.1	
Opening hours	6.2	1.2	
Waiting times	5.8	1.6	
Service provided by staff	6.7	0.8	
General rating for the health center	6.2	0.8	

Most of the participants rated the highest score on the location of the health center, the service provided by the staff, the opening hours, distance, and waiting times. For more details, see Table 2 and Suppl 1 Table 5.

### Variables related to the vaccination schedule

Roughly the same number of infants had come to the centers to receive each dose of the DTaP vaccine (25% for each of the four doses). Most of the respondents had their vaccination card with them (92.5%, *n* = 661) and knew when their next vaccination was scheduled. A minority of tutors were unable to vaccinate the infant in the past (10.5%, *n* = 75). For more details, see Table 3 and Suppl Table 6.

**Table 3:** Variables related to knowledge about the vaccination schedule

Variables	n	%	(95% CI)
**Dose scheduled for that day**			
2–6 months	598	83.6	(80.7–86.2)
18 months	117	16.4	(13.8–19.3)
**Has a vaccination card**			
Yes	661	92.5	(90.3–94.2)
No	54	7.5	(5.8–9.7)
**Knows the date of the next vaccination**			
Yes	602	84.2	(81.3–86.7)
No	113	15.8	(13.3–18.7)
***Reasons for ignoring the date of the next vaccination***			
*I was not told*	36	31.9	(23.9–41.1)
*I did not understand, or it was unclear*	13	11.5	(6.7–18.9)
*I do not remember*	47	41.6	(32.8–51.0)
*Other reasons*	17	15.0	(9.5–23.0)

### Variables related to vaccine usefulness, vaccination delays, and vaccine reticence

Most tutors identified health professionals (such as physicians and nurses) as their main source of vaccine information (66.6%, *n* = 476). Nearly all the tutors think that vaccines are useful for preventing or avoiding diseases (99.2%, *n* = 709). One-third of the tutors have previously delayed the infant's vaccination (29.8%, *n* = 213), reporting reasons such as location, opening hours, accessibility, and/or distance, fear of COVID-19, and problems affecting the infant or the interviewee. Only 26 participants had previously decided not to vaccinate the infant (3.6%), and most of them said that they were afraid of the risks and adverse effects of the vaccine. Finally, most of them (69.2%, *n* = 18) changed their mind and vaccinated him/her. For more details, see Table 4 and Suppl Table 7.

**Table 4:** Variables related to vaccine usefulness, vaccination delays and vaccine reticence

Variables	n	%	(95% CI)
**I know what vaccines are used for in general**			
I know what they are used for	709	99.2	(98.1–99.6)
I do not know what they are used for	6	0.8	(0.4–1.8)
**Sources of information accessed**			
Health professionals	476	66.6	(63.0–69.9)
Family and friends	102	14.3	(11.9–17.0)
Internet	137	19.2	(16.4–22.2)
**Has been unable to vaccinate the infant in the past**			
Has been able to do so	640	89.5	(87.0–91.5)
Has not been able to vaccinate the infant	75	10.5	(8.4–13.0)
***Reasons for being unable to vaccinate the infant***			
*Vaccination system issues*	65	86.7	(76.8–92.7)
*Problems of the infant or tutor*	10	13.3	(7.2–23.2)
***And if this has happened more than once***			
*Yes*	29	38.7	(28.2–50.3)
*No*	46	61.3	(49.7–71.7)
***Feelings linked to being unable to have the child vaccinated***			
*It is the way things are for me*	32	42.7	(31.8–54.2)
*I became upset*	25	33.3	(23.5–44.9)
*I did not mind*	18	24.0	(15.5–35.1)
**Prior delays in vaccination**			
No prior history of delays	502	70.2	(66.7–73.4)
Yes, with prior history of delays	213	29.8	(26.5–33.2)
***Reasons for prior delays***			
*Problems with the infant or tutor*	67	31.5	(25.5–38.0)
*Issues due to the healthcare centers' location, opening hours, accessibility, and/or distance*	75	35.2	(29.0–41.9)
*Fear or COVID-19*	71	33.3	(27.3–40.0)
**History of deciding not to vaccinate the infant**			
No	689	96.4	(94.7–97.5)
Yes	26	3.6	(2.5–5.3)
***Reasons for not vaccinating the infant***			
*Fear of vaccine risks*	17	65.4	(44.7–81.5)
*Fear of COVID-19*	5	19.2	(7.9–39.9)
*Expensive complementary vaccines*	4	15.4	(5.6–35.7)
***Whether the respondent changed his/her opinion regarding vaccination rejection***			
*Yes*	18	69.2	(48.4–84.4)
*No*	8	30.8	(15.6–51.6)

### Comparison between on-schedule and delayed participants

Most of the participants were on schedule (75%, *n* = 536), and in all cases, the main reason to be in the health center was vaccination. More participants enrolled in the public system were delayed. Most on-schedule participants attended a health center outside of their municipality of residence (56.5%, *n* = 303); in contrast, 54.8% (*n* = 98) of the delayed ones went to a health center located in their own municipality. Regarding transport, 54.7% (*n* = 293) of the on-schedule participants arrived by car, and most required less than 30 min to reach the health center; in contrast, 41.9% (*n* = 75) of the delayed ones arrived by car, and 38% (*n* = 68) on foot, while 72.1% (*n* = 129) of the delayed ones needed under 30 min. Of the on-schedule participants, 32.1% (*n* = 172) and 35.1% (*n* = 188) had come to receive the first and second dose, respectively; in contrast, 38.6% (*n* = 69) and 40.2% (*n* = 72) of the delayed ones had come to get the third and fourth dose. In both cases, most were aware of their next vaccination and were able to vaccinate the infant. With respect to information sources, 64.9% (*n* = 348) of the on-schedule participants talked to healthcare staff, compared to 71.5% (*n* = 128) of the delayed respondents. Of those who solved their questions on the Internet, 21.2% (*n* = 114) were on schedule, and 12.9% (*n* = 23) were late. Finally, regarding their history of vaccination delays, 13.4% (*n* = 72) of the on-schedule participants had not been delayed in the past; in contrast, 78.8% (*n* = 141) of the currently delayed respondents had been late in the past. In both cases, the main reasons not being delayed in the past were the location, opening hours, and/or distance. The main reasons for not being able to vaccinate the children were infant health problems (37.5%, *n* = 27) of the on-schedule participants, while fear of contracting COVID-19 at the health center was reported by 40.4% (*n* = 57) of the delayed ones. For more details, see Suppl. Table 8.

### The scale of Trust and Positive Attitudes Towards Vaccines

#### Dimensionality and Internal consistency

There was only one missing case for each item. The responses were skewed to the right, with a ceiling effect; that is, more than 15% of responses fell into the highest score in each item [35]. The parallel analysis revealed only one factor. Most of the items of this scale have factor loadings greater than 0.70, except for one (“I am satisfied with all the vaccines available on the vaccination calendar”), which had a factor loading of 0.58 and was thus removed from the scale. For more details, see Tables 5 and 6. The Internal consistency was high, α = 0.7918. See Table 7.

**Table 5:** Factor loadings of the items of the of the initial 6-item Trust and positive attitudes scale

Items	Factor 1	Uniqueness*
1	0.8512	0.2754
2	0.8435	0.2884
3	0.7598	0.4228
4	0.7848	0.3842
5	0.5841	0.6588
6	0.7217	0.4792

Note: *Uniqueness is the variance that is ‘unique’ to the variable and not shared with other variables. It is equal to 1 – communality (variance that is shared with other variables)

**Table 6:** Factor loadings of the items of the final 5-item Trust and positive attitudes scale (without item #5)

Items	Factor 1	Uniqueness
1	0.8614	0.2416
2	0.8677	0.2064
3	0.7787	0.3933
4	0.7456	0.4107
6	0.6947	0.4802

Note: *Uniqueness is the variance that is ‘unique’ to the variable and not shared with other variables. It is equal to 1 – communality (variance that is shared with other variables)

**Table 7:** Internal consistency of the final 5-item Scale Trust and positive attitudes

Items	Sign	Item-test correlation	Item-rest correlation	Average interitem covariance	Alpha
1	+	0.7839	0.6571	0.2077315	0.7287
2	+	0.7647	0.6360	0.2158446	0.7368
3	+	0.6931	0.5544	0.2402499	0.7624
4	+	0.7497	0.5713	0.2056293	0.7529
6	+	0.7573	0.5268	0.1949635	0.7861
Test scale				0.2128838	0.7918

### Associations

With respect to the sociodemographic variables examined, being a caregiver other than the mother increased the risk of vaccination delays (OR = 4,39; 95% CI: 1.49–12.89; *p* = 0.007). Similarly, having more than one child increases the risk (OR = 1.6; 95% CI: 1.12–2.39; *p* = 0.010). See Table 8.

**Table 8:** Univariate and multivariate association in relation to the delay in vaccination: Sociodemographic variables

Domain 1: Sociodemographic variables				
	Model 0		Model 1	
Variables	OR (95% CI)	*p*-value	OR (95% CI)^a^	*p*-value
Tutor´s sex				
Woman	1		1	
Man	1.48 (0.97–2.26)	0.071	1.22 (0.25–5.97)	0.804
Child´s sex				
Woman	1		1	
Man	0.78 (0.56–1.10)	0.161	0.71 (0.49–1.01)	0.054
Relationship between the tutor and the infant				
Mother	1		1	
Father	1.64 (1.06–2.53)	0.027	1.83 (0.36–9.26)	0.466
Other	5.11 (1.79–2.14)	0.002	**4.39 (1.49–12.89)**	**0.007**
Tutor´s marital status				
Married	1		1	
Single	1.34 (0.91–1.96)	0.135	1.25 (0.82–1.89)	0.298
Separated/divorced	2.79 (0.60–12.90)	0.190	3.15 (0.64–15.53)	0.158
Family structure				
Single parent	1		1	
Biparental and/or extended	0.66 (0.40–1.12)	0.123	0.88 (0.50–1.54)	0.651
Wealth of neighborhood where they live		0.2855^b^		
High-income	1		-	
Medium-income	0.73 (0.48–1.08)			
Low-income	0–84 (0.55–1.28)			
Type of health insurance of the tutor and infant				
Public	1		1	
Private	0.61 (0.43–0.87)	0.007	0.69 (0.44–1.08)	0.107
Tutor´s nationality				
Chilean	1		1	
Other	1.29 (0.92–1.82)	0.135	1.13 (0.75–1.70)	0.571
Tutor´s education		0.0392^b^	0.1722^b^	
Non higher education	1		1	
Incomplete higher education	1.28 (0.75–2.19)		1.72 (0.97–3.07)	
Complete higher education	0.69 (0.47–1.01)		0.96 (0.62–1.50)	
Postgraduate studies	0.50 (0.18–1.38)		0.86 (0.29–2.54)	
Tutor´s labor status				
Employed	1		-	
Unemployed	1.03 (0.67–1.60)	0.895		
Number of children of the mother				
One child	1		1	
More than one child	1.51 (1.07–2.13)	0.020	**1.64 (1.12–2.39)**	**0.010**

Notes:

^a^ The multivariable model included all variables within the same Domain. ^b^ This p-value refers to the Walt test

The dyads attending the healthcare center for a reason other than only receiving a vaccine were less likely to delay vaccination. See Table 9.

**Table 9:** Univariate and multivariate association in relation to the delay in vaccination: Health center features

Domain 2: Health center features				
	Model 0		Model 1	
Variables	OR (95% CI)	*p*-value	OR (95% CI)^a^	*p*-value
Main reason for bringing the infant to this center				
To vaccinate the infant	1		1	
**Another reason**	**0.22 (0.07–0.71)**	**0.012**	**0.25 (0.08–0.83)**	**0.025**
The health center belongs to the tutor's municipality of residence				
Belongs to the municipality	1		1	
Does not belong to the municipality	0.64 (0.45–0.89)	0.009	0.70 (0.48–1.04)	0.073
Means of transportation to travel to the health center				
Personal transport	1			
Public transport	1.13 (0.73–1.72)	0.587	-	
Time spent traveling to the healthcare center today				
< 30 min	1		1	
30 or more minutes	0.76 (0.52–1.10)	0.149	0.95 (0.62–1.46)	0.826

The dose scheduled for the 18th month (OR = 6.79; 95% CI: 4.40–10.48; *p* < 0.001) was associated with a greater risk of delays. See Table 10.

**Table 10:** Univariate and multivariate association in relation to the delay in vaccination: Variables related to knowledge about vaccination schedule

Domain 3: Variables related to knowledge about vaccination schedule				
	Model 0		Model 1	
Variables	OR (95% CI)	*p*-value	OR (95% CI)^a^	*p*-value
Dose scheduled for that day				
2–6 months	1		1	
**18 months**	**7.34 (4.79–11.25)**	** < 0.001**	**6.79 (4.40–10.48)**	** < 0.001**
Has a vaccination card				
Yes	1		1	
No	0.50 (0.23–1.08)	0.077	0.53 (0.23–1.21)	0.133
Knows the date of the next vaccination				
Yes	1		1	
No	2.01 (1.31–3.98)	0.001	1.50 80.17–0.27)	0.095

Having a history of vaccination delays increased the risk of delaying subsequent vaccinations (OR = 24.58; 95% CI: 15.75–38.64; *p* < 0.001), and using the internet as a source of information reduced the risk for delay compared with getting the information from health professionals. See Table 11.

**Table 11:** Univariate and multivariate association in relation to the delay in vaccination: Variables related to vaccine usefulness, vaccination delays, and vaccine reticence

Domain 4: Variables related to vaccine usefulness, vaccination delays, and vaccine reticence				
	Model 0		Model 1	
Variables	OR (95% CI)	*p*-value	OR (95% CI)^a^	*p*-value
I know what vaccines are used for…				
I know	1			
I do not know	1.50 (0.27–8.27)	0.640	-	
Sources of information accessed				
Health professionals	1		1	
Family and friends	1.03 (0.64–1.66)	0.908	1.69 (0.90–3.18)	0.106
**Internet**	**0.55 (0.33–0.90)**	**0.017**	**0.53 (0–29-0.98)**	**0.043**
Been able to vaccinate the infant in the past				
No	1		1	
Yes	0.52 (0.31–0.86)	0.010	0.77 (0.40–1.48)	0.438
Prior delays in vaccination				
No history of prior delays	1		1	
**Yes, prior history of delays**	**23.91 (15.46–36.97)**	** < 0.001**	**24.59 (15.65–38.64)**	** < 0.001**
History of deciding not to vaccinate the infant				
No	1		1	
Yes	2.68 (1.21–5.90)	0.015	1.32 (0.48–3.67)	0.590

Finally, the greater a respondent's trust in vaccination and positive attitudes towards it, the lower the risk of delays (OR = 0.67; 95% CI: 0.49–0.92; *p*-value = 0.013). For more details, see Table 12.

**Table 12:** Univariate and multivariate association in relation to the delay in vaccination: Trust and Positive Attitudes Towards Vaccines Scale

Domain 5: Trust and Positive Attitudes Towards Vaccines Scale				
	Model 0		Model 1	
Variables	OR (95% CI)	*p*-value	OR (95% CI)^a^	*p*-value
**Trust and positive attitudes**	**0.68 (0.50–0.93)**	**0.016**	**0.67 (0.49–0.92)**	**0.013**
Relationship between the tutor and the infant				
Mother	1		1	
**Father**	**1.64 (1.06–2.53)**	**0.027**	**1.83 (1.17–2.88)**	**0.008**
**Other**	**5.11 (1.79–2.14)**	**0.002**	**4.40 (1.51–12.83)**	**0.007**
Number of children of the mother				
One child	1		1	
More than one child	1.51 (1.07–2.13)	0.020	**1.69 (1.17–2.41)**	**0.005**

## Discussion

This is the first Latin American study during the pandemic aimed to validate an instrument to evaluate the factors associated with vaccination delays in the administration of the DTaP vaccine. Trust in vaccination and positive attitudes toward it constituted one of the main variables that reduced the risk of delays in the population studied. Other factors that reduced delays were attending the healthcare center for a reason other than only vaccination and obtaining information about vaccines from the Internet. On the other hand, the main risk factors for delaying vaccination were having a prior history of delay, having more than one child, and that the child attends to the health center with adults other than the mother.

Trust and attitudes have also been studied in connection with other vaccines. For instance, in 2017, an article on Canadian parents' acceptance of the vaccine revealed that those with a high level of trust in the physicians working in the healthcare system were more likely to have a strong intention of vaccinating their children [36]. The article also showed that parents' positive attitudes were associated with a stronger intention of vaccinating their children [36]. In a similar manner, a systematic review of the reasons and factors associated with vaccination delays stressed the relevance of parents' attitudes and how insufficient knowledge, fear of adverse effects, the belief that vaccines are ineffective, and the belief that healthcare systems are not wholly trustworthy affected the vaccination delay [37]. Other studies have found that low treatment quality in public clinics was one of the strongest predictors of a delayed first immunization [38]. In our study, there was a strong univariate association between vaccination delay and living in a different municipality from the location of the health center, and tutors said that the main reason for this behavior was the quality of the service. Although this variable was no longer significant in the multivariate analyses (*p* = 0.074), it highlights the importance of quality of service to build trust in the vaccination process.

In addition, people who actively seek health information are more involved in making decisions about their healthcare and tend to experience improved health outcomes and reduced healthcare expenses [39]. This may explain why tutors who obtain online information are less likely to exhibit delays. Parents usually seek health information online regarding diseases and treatments and feel that the Internet is a good resource to do so [40]. This finding highlights the importance of keeping digital and non-digital media communication media up-to-date and stocked with trustworthy information for users to make their own decisions based on reliable information [41]. Vaccine history has long been plagued by the spread of unscientific information ─such as the association between the use of thimerosal as a vaccine component and the rise in autism in children [42, 43] ─which has altered families' behavior regarding vaccines. Several national state agencies in Chile, such as the Ministry of Health and the Ministry of Social Development, provide reliable health information to the Chilean population.

Several factors were found to be linked to increases in the risk of delays, such as the tutor being a person other than the mother accompanying the infant, having more than one child, and having to receive the 18-month dose of the vaccine. These findings are similar to those from a recent study in South Africa, where the main reasons for vaccination delays were the greater age of the child, the greater number of children, and paternal and maternal unemployment [44]. Additionally, we also found that having a history of delays also exhibited an increased risk of later delays. For instance, in 2013, a study on the reasons for non-fulfillment and delays in the primary vaccination schedule indicated that the main causes for these issues were parents' fear of adverse reactions, prior delays in vaccination, and the absence of the relevant vaccine at the health center [45]. In addition, the fact that delays can be caused by the mother being unavailable to take her children to the health center or having more than one child hints at insufficient family support or socio-economic difficulties in providing the basic care that young children require, such as vaccinations. The state should provide help to deprived families to facilitate the fulfillment of these basic care children need.

### Limitations

One of the difficulties that we encountered was the pandemic-derived context in which the study was conducted. This meant that none of the healthcare centers in Chile could operate normally, with reduced staff and shorter opening hours compared to the pre-pandemic situation. For example, during the data collection, the sanitary conditions and people's fear of contracting Covid-19 resulted in considerably decreased attendance and a reduced sample size. Therefore, the results presented must be evaluated with caution and compared with future research on the same population after the pandemic is over.

The strike that affected all public healthcare centers ─due to healthcare personnel demands for improved working conditions during the pandemic─ was another difficult situation for the research team to handle. To deal with this setback, we extended the data collection process.

Regarding the instrument, we identified some questions that had provided far too many answer choices. Since this made the initial analysis unnecessarily complicated, we reduced the answer choices/options to two or, at most, three to facilitate our analyses. In addition, we removed the open-ended questions originally included because only a very small number of participants answered these questions. Finally, regarding the whole questionary, and particularly with the Trust and Positive Attitudes Towards Vaccines Scale, we are not providing a complete account of the validity of this instrument or an extensive evaluation of the psychometric properties as suggested by other authors [35]. We did provide information about the content validity done by experts in the field, internal consistency, construct validity using EFA, and the presence of floor and ceiling effects. However, future research is necessary to explore the reproducibility of the results, the responsiveness or the ability to detect important changes over time in the concept measured, how to interpret the results to assign qualitative meaning to quantitative scores [35], and how to reduce the ceiling effect found on the items of this scale.

## Conclusions

Our results highlight the relevance of offering adequate and trustworthy information for users to be able to make good decisions. Aspects such as the users' ability to understand the information provided (in terms of language and knowledge about health), the quality of the care received, and the diversity of the services available at a healthcare center affect the acceptability of the interventions delivered and as well as vaccination levels [46]. All these features are valued by Chilean people and foreigners living in Chile.

In this study, we have developed an instrument that is easy to administer, straightforward to understand and requires little time to complete. Furthermore, the Trust and Positive Attitudes Towards Vaccines Scale is reliable and has a good item structure.

Further research is necessary for replicating these results beyond the pandemic-derived context. All things considered, the instrument presented in this article should help the scientific community to evaluate future interventions aimed at increasing trust and positive attitudes toward the vaccination process.

### Supplementary Information

**Additional file 1. **Supplementary tables 1-9

**Additional file 2. **Annex: Variables include in the questionnaire

## Data Availability

The datasets used and/or analyzed during the current study are available from the corresponding author upon reasonable request.

## References

[1] Salud OMdl. Cobertura vacunal. 2021. https://www.who.int/es/news-room/fact-sheets/detail/immunization-coverage

[2] World Health Organization (2020). Principios rectores para las actividades de inmunización durante la pandemia de COVID-19.

[3] Vasudevan L, Labrique AB, Mehra S, Wu L, Levine O, Feikin D (2014). Maternal determinants of timely vaccination coverage among infants in rural Bangladesh. Vaccine.

[4] Le Polain de Waroux O, Schellenberg JR, Manzi F, Mrisho M, Shirima K, Mshinda H (2013). Timeliness and completeness of vaccination and risk factors for low and late vaccine uptake in young children living in rural southern Tanzania. Int Health..

[5] Glanz JM, Newcomer SR, Narwaney KJ, Hambidge SJ, Daley MF, Wagner NM (2013). A population-based cohort study of undervaccination in 8 managed care organizations across the United States. JAMA Pediatr.

[6] Hu Y, Chen Y, Guo J, Tang X, Shen L (2014). Completeness and timeliness of vaccination and determinants for low and late uptake among young children in eastern China. Hum Vaccin Immunother.

[7] Dombkowski KJ, Lantz PM, Freed GL (2002). The need for surveillance of delay in age-appropriate immunization. Am J Prev Med.

[8] Tippins A, Leidner AJ, Meghani M, Griffin A, Helgenberger L, Nyaku M (2017). Timeliness of childhood vaccination in the Federated States of Micronesia. Vaccine.

[9] Wang Z, Rost G, Moghadas SM (2019). Delay in booster schedule as a control parameter in vaccination dynamics. J Math Biol.

[10] Salud OMdl (2011). Plan de acción mundial sobre vacunas 2011–2020.

[11] Opel DJ, Mangione-Smith R, Taylor JA, Korfiatis C, Wiese C, Catz S (2011). Development of a survey to identify vaccine-hesitant parents: the parent attitudes about childhood vaccines survey. Hum Vaccin.

[12] Opel DJ, Taylor JA, Mangione-Smith R, Solomon C, Zhao C, Catz S (2011). Validity and reliability of a survey to identify vaccine-hesitant parents. Vaccine.

[13] Cunningham RM, Kerr GB, Orobio J, Munoz FM, Correa A, Villafranco N (2019). Development of a Spanish version of the parent attitudes about childhood vaccines survey. Hum Vaccin Immunother.

[14] Napolitano F, D'Alessandro A, Angelillo IF (2018). Investigating Italian parents' vaccine hesitancy: A cross-sectional survey. Hum Vaccin Immunother.

[15] Abd Halim H, Abdul-Razak S, Md Yasin M, Isa MR (2020). Validation study of the Parent Attitudes About Childhood Vaccines (PACV) questionnaire: the Malay version. Hum Vaccin Immunother.

[16] Organización Panamericana de la Salud (2014). Metodología para la evaluación de oportunidades perdidas de vacunación.

[17] Dube E, Gagnon D, Nickels E, Jeram S, Schuster M (2014). Mapping vaccine hesitancy–country-specific characteristics of a global phenomenon. Vaccine.

[18] Schuster M, Eskola J, Duclos P, SAGE Working Group on Vaccine Hesitancy (2015). Review of vaccine hesitancy: Rationale, remit and methods. Vaccine..

[19] World Health Organization (2020). Immunization agenda 2030. A global strategy to leave no one behind.

[20] Infectología SCd. Revista Chilena de Infectología Marzo. 2020;37(Cobertura y oportunidad de vacunación contra la difteria-tétanos-pertussis en Chile, 2013–2017; un análisis de Kaplan-Meier utilizando el Registro Nacional de Inmunizaciones):29–33.

[21] Bastías M, Brstilo I, González C (2021). Vacunación programática 2020 en Chile en tiempos de pandemia por SARS-CoV-2. Rev Chilena Infectol.

[22] Larson HJ, Jarrett C, Schulz WS, Chaudhuri M, Zhou Y, Dube E (2015). Measuring vaccine hesitancy: The development of a survey tool. Vaccine.

[23] Tabachnick BG, Fidell LS (2013). Using Multivariate Statistics: Pearson New International Edition: Pearson Education.

[24] Cramm JM, Nieboer AP (2018). Validation of an instrument for the assessment of patient-centred care among patients with multimorbidity in the primary care setting: the 36-item patient-centred primary care instrument. BMC Fam Pract.

[25] Nunnally JC. Psychometric theory. New York: McGraw-Hill; 1978.

[26] Watkins MW (2018). Exploratory Factor Analysis: A Guide to Best Practice. J Black Psychol.

[27] Fabrigar L, Wegener D, MacCallum R, Strahan E (1999). Evaluating the Use of Exploratory Factor Analysis in Psychological Research. Psychological Methods..

[28] Costello A, Osborne J (2005). Best practices in exploratory factor analysis: four recommendations for getting the most from your analysis. Pract Assess Res Eval.

[29] Horn JL (1965). A rationale and test for the number of factors in factor analysis. Psychometrika.

[30] Timmerman ME, Lorenzo-Seva U (2011). Dimensionality assessment of ordered polytomous items with parallel analysis. Psychol Methods.

[31] Ledesma RD, Valero-Mora P. Determining the Number of Factors to Retain in EFA: An easy-to-use computer program for carrying out Parallel Analysis. Pract Assess Res Eval. 2019;12(2):1–11.

[32] Lloret-Segura S, Ferreres-Traver A, Hernández-Baeza A, Tomás-Marco I. El análisis factorial exploratorio de los ítems: una guía práctica, revisada y actualizada. Anales de Psicología. 2014;30(3):1151–69.

[33] Cronbach LJ (1951). Coefficient alpha and the internal structure of tests. Psychometrika.

[34] Kline P. Handbook of Psychological Testing. London: Routledge; 2000.

[35] Bot SDM, Terwee CB, van der Windt DAWM, Bouter LM, Dekker JM, de Vet HCW. Psychometric evaluation of selfreport questionnaires: the development of a checklist. In Adér HJ, Mellenberg C, editors. Proceedings of the second workshop on research methodology. Amsterdam: VU University; 2003. p. 161–68.

[36] Dube E, Gagnon D, Ouakki M, Bettinger JA, Witteman HO, MacDonald S (2018). Measuring vaccine acceptance among Canadian parents: A survey of the Canadian Immunization Research Network. Vaccine.

[37] Rainey JJ, Watkins M, Ryman TK, Sandhu P, Bo A, Banerjee K (2011). Reasons related to non-vaccination and under-vaccination of children in low and middle income countries: findings from a systematic review of the published literature, 1999–2009. Vaccine.

[38] Feemster KA, Spain CV, Eberhart M, Pati S, Watson B (2009). Identifying infants at increased risk for late initiation of immunizations: maternal and provider characteristics. Public Health Rep.

[39] Greene J, Hibbard JH, Sacks R, Overton V, Parrotta CD (2015). When patient activation levels change, health outcomes and costs change, too. Health Aff (Millwood).

[40] Harvey S, Memon A, Khan R, Yasin F (2017). Parent's use of the Internet in the search for healthcare information and subsequent impact on the doctor-patient relationship. Ir J Med Sci.

[41] Yudianto B, Caldwell PH, Nanan R, Barnes EH, Scott KM (2023). Patterns of parental online health information-seeking behaviour. J Paediatr Child Health.

[42] Geier MR, Geier DA (2003). Neurodevelopmental disorders after thimerosal-containing vaccines: a brief communication. Exp Biol Med (Maywood).

[43] Redwood L, Bernard S, Brown D (2001). Predicted mercury concentrations in hair from infant immunizations: cause for concern. Neurotoxicology.

[44] Mthiyane TN, Cohen C, Norris SA, Walaza S, Tempia S, Cohen AL (2019). Factors associated with missed and delayed DTP3 vaccination in children aged 12–59 months in two communities in South Africa, 2012–2013. S Afr Med J.

[45] V. C-RE, Aarón P-R. Causas de incumplimiento y retraso del esquema primario de vacunación en niños atendidos en el Hospital Infantil de México “Federico Gómez”. Aten Farm. 2013;20:6–11.

[46] MacDonald NE, SAGE Working Group on Vaccine Hesitancy (2015). Vaccine hesitancy: Definition, scope and determinants. Vaccine..

