# Supplementary material for: Delays in the vaccination of infants between 2 and 18 months of age: associated factors in Chile
Source: BMC Public Health. 2023 Sep 28;23:1882. doi: 10.1186/s12889-023-16769-3 (PMC10540413; doi:10.1186/s12889-023-16769-3)
Supplement: Supplementary file 1 — Additional file 1. Supplementary tables 1-9 [file 12889_2023_16769_MOESM1_ESM.docx]

Table 1: Distribution of participation by vaccination.

| Variables | n | % |
| --- | --- | --- |
| CESFAM Cruz Melo (public) | 84 | 11.8 |
| CESFAM Domeyko (public) | 151 | 21.1 |
| CEFAM N°1 (public) | 98 | 13.7 |
| Clínica Dávila (private) | 151 | 21.1 |
| Clínica Indisa (public) | 114 | 15.9 |
| Clínica Santa María (public) | 117 | 16.4 |

Table 2: Sociodemographic characteristics of the infant and the tutor.

| Variable | | n | % |
| --- | --- | --- | --- |
| **Sex of the tutor** | | | |
| Woman | 591 | | 82.7 |
| Man | 124 | | 17.3 |
| **Sex of the infant** | | | |
| Woman | 347 | | 48.5 |
| Man | 368 | | 51.5 |
| **Relationship between the tutor and the infant** | | | |
| Mother | 586 | | 82 |
| Father | 114 | | 15.9 |
| Grandmother | 7 | | 1.0 |
| Uncle | 7 | | 1.0 |
| Great-grandmother | 1 | | 0.1 |
| **Marital status** | | | |
| Married | 210 | | 29.4 |
| Single | 491 | | 68.6 |
| Separated/Divorced | 7 | | 1.0 |
| Civil union | 7 | | 1.0 |
| **Family structure** | | | |
| Biparental | 626 | | 87.6 |
| Single parent | 74 | | 10.3 |
| Extended family | 15 | | 2.1 |
| **Municipality of residency of the tutor** |  | |  |
| Santiago | 267 | | 37.3 |
| Independencia | 102 | | 14.3 |
| Other municipality | 346 | | 48.4 |
| **Nationality of tutor** | | | |
| Chile | 379 | | 53.0 |
| Venezuela | 161 | | 22.5 |
| Perú | 86 | | 12.0 |
| Colombia | 39 | | 5.5 |
| Other | 50 | | 7.0 |
| **Nationality of infant** | | | |
| Chile | 704 | | 98.5 |
| Venezuela | 6 | | 0.8 |
| Perú | 4 | | 0.6 |
| Spain | 1 | | 0.1 |
| **Type of health insurance of the tutor and infant** | | | |
| Fonasa | 405 | | 56.6 |
| Isapre | 291 | | 40.7 |
| Particular | 19 | | 2.7 |
| **Number of children of the mother** |  | |  |
| One child | 459 | | 64.2 |
| More than one child | 256 | | 35.8 |

Table 3: Educational level and occupation of the respondent and the main breadwinner

| Educational level | Of the respondent | | Main breadwinner | | | |
| --- | --- | --- | --- | --- | --- | --- |
|  | **n** | **%** | | **n** | **%** |  |
| No formal studies | 0 | 0 | | 0 | 0 |  |
| Incomplete Basic; incomplete high school or elementary school | 4 | 0.6 | | 1 | 0.1 |  |
| Full Basic; complete elementary or high school | 8 | 1.1 | | 11 | 1.5 |  |
| Incomplete media or professional technician | 29 | 4.1 | | 26 | 3.7 |  |
| Half or complete professional technician | 172 | 24.1 | | 170 | 23.8 |  |
| Incomplete technical institute or professional institute | 22 | 3.1 | | 17 | 2.4 |  |
| Complete technical institute or professional institute | 97 | 13.3 | | 96 | 13.2 |  |
| Incomplete university | 64 | 9.0 | | 65 | 9.1 |  |
| Complete university | 289 | 40.5 | | 303 | 42.5 |  |
| Postgraduate | 30 | 4.2 | | 26 | 3.7 |  |
| Retired | 0 | 0 | | 0 | 0 |  |

| Occupation | Of the respondent | | | Main breadwinner | |  |
| --- | --- | --- | --- | --- | --- | --- |
|  | **n** | **%** | **n** | | **%** | |
| Unskilled workers in sales and services, agricultural laborers, forestry workers, construction workers, etc. | 66 | 9.2 | 94 | | 13.2 | |
| Workers, operators, and artisans of mechanical arts and other trades | 36 | 5.0 | 109 | | 15.3 | |
| Service workers and vendors in commerce and markets | 111 | 15.6 | 115 | | 16.2 | |
| Farmers and skilled agricultural and fishing workers | 0 | 0 | 2 | | 0.3 | |
| Plant and machine operators and vehicle fitters/drivers | 5 | 0.6 | 21 | | 3.0 | |
| Public and private office employees | 105 | 14.7 | 95 | | 13.3 | |
| Professionals, scientists, and intellectuals | 194 | 27.2 | 224 | | 31.3 | |
| Senior executive (general manager or area or sector manager) of private or public companies. Director or owner of large companies | 5 | 0.6 | 8 | | 1.1 | |
| Other unidentified groups (includes rentiers, disabled, etc.) | 63 | 8.9 | 17 | | 2.1 | |
| Currently, without paid work (Housewives, unemployed) | 130 | 18.2 | 30 | | 4.2 | |
| Retired | 0 | 0 | 0 | | 0 | |

Table 4: Characteristics related to the choice of the health center.

| Variable | n | % |
| --- | --- | --- |
| **The main reason for bringing the infant to this center** | | |
| To vaccinate the infant | 673 | 94.1 |
| Health check | 30 | 4.2 |
| Another reason | 12 | 1.7 |
| **The vaccination center location regarding residence** | | |
| Yes | 331 | 46.3 |
| No | 384 | 53.7 |
| ***For those who go to the health center not located in the municipality of residence, the reasons:*** | | |
| Quality of care | 215 | 56.00 |
| Health insurance | 69 | 18.00 |
| Closeness | 54 | 14.1 |
| Use of health center for other health issues | 46 | 12.0 |
| **Means of transport used to travel to this health care center** | | |
| Car | 368 | 51.5 |
| Walking | 213 | 29.8 |
| Micro | 45 | 6.3 |
| Subway | 25 | 3.5 |
| Cab | 40 | 5.6 |
| “Colectivo” | 24 | 3.4 |
| **Time spent traveling to the health care center today** | | |
| Less than 30 minutes | 484 | 67.7 |
| From 30 minutes to less than 1 hour | 170 | 23.8 |
| 1 to 2 hours | 59 | 8.3 |
| Greater than 2 hours | 2 | 0.3 |

Table 5. Rating of several aspects of the vaccination center (1 to 7)

|  | 1 | | 2 | | 3 | | 4 | | 5 | | 6 | | 7 | |
| --- | --- | --- | --- | --- | --- | --- | --- | --- | --- | --- | --- | --- | --- | --- |
|  | **n** | **%** | **n** | **%** | **n** | **%** | **n** | **%** | **n** | **%** | **n** | **%** | **n** | **%** |
| Location | 5 | 0.7 | 1 | 0.1 | 6 | 0.8 | 22 | 3.1 | 89 | 12.3 | 158 | 22.3 | 434 | 60.7 |
| Accessibility and distance | 4 | 0.5 | 6 | 0.8 | 15 | 2.1 | 25 | 3.5 | 114 | 15.9 | 160 | 22.4 | 391 | 54.7 |
| Opening hours | 7 | 1.0 | 4 | 0.6 | 18 | 2.5 | 44 | 6.2 | 92 | 12.9 | 123 | 17.2 | 427 | 59.7 |
| Waiting times | 31 | 4.4 | 17 | 2.2 | 35 | 4.9 | 47 | 6.6 | 85 | 11.9 | 152 | 21.3 | 348 | 48.7 |
| Service provided by staff | 3 | 0.4 | 1 | 0.1 | 4 | 0.6 | 10 | 1.4 | 36 | 5.0 | 95 | 13.3 | 566 | 79.2 |

Table 6: Characteristics related to the vaccination schedule.

| Variable | n | % |
| --- | --- | --- |
| **Dose scheduled for that day** | | |
| 1st dose (2 months) | 186 | 26.0 |
| 2nd dose (4 months) | 212 | 29.7 |
| 3rd dose (6 months) | 200 | 28.0 |
| 4th dose (18 months) | 117 | 16.4 |
| **Calculation of up-to-date vaccination concerning the date of the interview with the date of birth** | | |
| You are vaccination up to date | 536 | 75.0 |
| You are overdue for your vaccination | 179 | 25.0 |
| **Has a vaccination card** | | |
| Yes | 661 | 92.5 |
| No | 54 | 7.6 |
| **Knows the date of the next vaccination** | | |
| Yes | 602 | 84.2 |
| No | 113 | 15.8 |
| **Reasons for ignoring the date of the next vaccination** |  |  |
| I was not told | 36 | 31.9 |
| I did not understand, or it was unclear | 13 | 11.5 |
| I do not remember | 47 | 41.6 |
| Other reasons | 17 | 15.0 |
| **Has been unable to vaccinate the infant in the past** | | |
| Has been able to do so | 75 | 10.5 |
| Has not been able to vaccinate the infant | 640 | 89.5 |
| **Reasons for being unable to vaccinate the infant** | | |
| Vaccination system issues | 65 | 86.7 |
| Problems of the infant or tutor | 10 | 13.3 |
| **And if this has happened more than once** | | |
| Yes | 29 | 38.7 |
| No | 46 | 61.3 |
| **Feelings linked to being unable to have the child vaccinated** | | |
| It is the way things are for me | 32 | 42.7 |
| I became upset | 25 | 33.3 |
| I did not mind | 18 | 24.0 |

Table 7: Characteristics related to vaccination delays and vaccine hesitancy.

| Variable | n | | % |
| --- | --- | --- | --- |
| **I know what vaccines are used for in general** | | | |
| I know what they are used for | 6 | 0.8 | |
| I do not know what they are used for | 709 | 99.2 | |
| **Sources of information accessed** | | | |
| Health professionals | 476 | | 66.6 |
| Friends | 102 | | 14.3 |
| Internet | 137 | | 19.2 |
| **Prior delays in vaccination** | | | |
| History of prior delays | 213 | | 29.8 |
| No history of prior delays | 502 | | 70.2 |
| **Reasons for prior delays** | | | |
| Problems with the infant or tutor | 75 | | 35.2 |
| Issues due to the vaccination centers' location, opening hours, accessibility, or distance | 71 | | 33.3 |
| Fear of COVID-19 | 67 | | 31.5 |
| **History of deciding not to vaccinate the infant** | | | |
| No | 26 | | 3.6 |
| Yes | 689 | | 96.4 |
| **Reasons for not vaccinating the infant** | | | |
| Fear of vaccine risks | 17 | | 65.4 |
| Fear of COVID-19 | 5 | | 19.2 |
| Expensive complementary vaccines | 4 | | 15.4 |
| **Whether the respondent changed his/her opinion regarding vaccination rejection** | | | |
| Yes | 18 | | 69.2 |
| No | 8 | | 30.8 |

Table 8: Comparison between up-to-date participants with those who are delayed.

|  | Up-to-date | | Delayed | |
| --- | --- | --- | --- | --- |
|  | **n** | **%** | **n** | **%** |
| Total | 536 | 75.0 | 179 | 25.0 |
| **SOCIODEMOGRAPHIC CHARACTERISTICS** | | | | |
| **Sex of the tutor** | | | | |
| Woman | 451 | 81.1 | 140 | 78.2 |
| Man | 85 | 15.9 | 39 | 21.8 |
| **Sex of the infant** | | | | |
| Woman | 252 | 47.0 | 95 | 53.1 |
| Man | 284 | 53.0 | 84 | 46.9 |
| **Relationship between the tutor and the infant** | | | | |
| Mother | 453 | 84.5 | 133 | 74.3 |
| Father | 77 | 14.4 | 37 | 20.7 |
| Grandmother | 2 | 0.4 | 5 | 2.8 |
| Uncle | 3 | 0.6 | 4 | 2.2 |
| Great-grandmother | 1 | 0.2 | 0 | 0.0 |
| **Marital status** | | | | |
| Married | 164 | 30.6 | 46 | 25.7 |
| Single | 361 | 67.4 | 130 | 72.6 |
| Separated/Divorced | 4 | 0.8 | 3 | 1.7 |
| Civil union | 7 | 1.3 | 0 | 0.0 |
| **Family structure** | | | | |
| Biparental | 474 | 88.4 | 152 | 84.9 |
| Nonparental | 12 | 2.2 | 3 | 1.7 |
| Extended | 50 | 9.3 | 24 | 13.4 |
| **Type of health insurance of the tutor and infant** | | | | |
| Fonasa | 288 | 53.7 | 117 | 65.4 |
| Isapre | 238 | 44.4 | 53 | 29.6 |
| Particular | 10 | 1.9 | 9 | 5.0 |
| **CHARACTERISTICS RELATED TO THE VACCINATION CENTER** | | | | |
| **The main reason for bringing the infant to this center** | | | | |
| To vaccinate the infant | 497 | 92.7 | 176 | 98.3 |
| Health check | 29 | 5.4 | 1 | 0.6 |
| Another reason | 10 | 1.9 | 2 | 1.1 |
| **The vaccination center location regarding residence** | | | | |
| Yes | 233 | 43.5 | 98 | 54.8 |
| No | 303 | 56.5 | 81 | 45.3 |
| ***For those who go to the health center not located in the municipality of residence, the reasons:*** | | | | |
| Quality of care | 168 | 55.5 | 47 | 58.0 |
| Health insurance | 56 | 18.5 | 13 | 16.1 |
| Closeness | 42 | 13.9 | 12 | 14.8 |
| Use of health center for other health issues | 37 | 12.2 | 9 | 11.1 |
| **Means of transport used to travel to this health care center** | | | | |
| Car | 293 | 54.7 | 75 | 41.9 |
| Walking | 145 | 27.1 | 68 | 38.0 |
| Micro | 25 | 4.7 | 20 | 11.2 |
| Subway | 21 | 3.9 | 4 | 2.2 |
| Cab | 34 | 6.3 | 6 | 3.4 |
| Colectivo | 18 | 3.4 | 8 | 3.4 |
| **Time spent traveling to the health care center today** | | | | |
| Less than 30 minutes | 355 | 66.2 | 129 | 72.1 |
| From 30 minutes to less than 1 hour | 135 | 25.2 | 35 | 19.6 |
| 1 to 2 hours | 45 | 8.4 | 14 | 7.8 |
| Greater than 2 hours | 1 | 0.2 | 1 | 0.6 |
| **Characteristics related to vaccination history and opinions** | | | | |
| **Dose scheduled for that day** | | | | |
| 1° dose (2 months) | 172 | 32.1 | 14 | 7.8 |
| 2° dose (4 months) | 188 | 35.1 | 24 | 13.4 |
| 3° dose (6 months) | 131 | 24.4 | 69 | 38.6 |
| 4° dose (18 months) | 45 | 8.4 | 72 | 40.2 |
| **Has a vaccination card** | | | | |
| Yes | 490 | 91.4 | 171 | 95.5 |
| No | 46 | 8.6 | 8 | 4.5 |
| **Knows the date of the next vaccination** | | | | |
| Yes | 465 | 86.8 | 137 | 76.5 |
| No | 71 | 13.3 | 42 | 23.5 |
| **It has happened that you have not been able to vaccinate the infant** | | | | |
| Sí | 47 | 8.8 | 28 | 15.6 |
| No | 489 | 91.2 | 151 | 84.4 |
| **Reasons for being unable to vaccinate the infant** | | | | |
| Vaccination system issues | 41 | 87.2 | 24 | 85.7 |
| Problems of the infant or tutor | 6 | 12.8 | 4 | 14.3 |
| **And if this has happened more than once** | | | | |
| Yes | 18 | 38.3 | 11 | 39.3 |
| No | 29 | 61.7 | 17 | 60.7 |
| **Feelings linked to being unable to have the child vaccinated** | | | | |
| It is the way things are for me | 18 | 38.3 | 14 | 50.0 |
| I became upset | 16 | 34.0 | 9 | 32.1 |
| I did not mind | 13 | 27.7 | 5 | 17.9 |
| **CHARACTERISTICS RELATED TO VACCINATION DELAYS AND VACCINE HESITANCY** | | | | |
| **Sources of information accessed** | | | | |
| Health professionals | 348 | 64.9 | 128 | 71.5 |
| Friends | 74 | 13.8 | 28 | 15.6 |
| Internet | 114 | 21.2 | 23 | 12.9 |
| **I know what vaccines are used for in general** | | | | |
| I know what they are used for | 532 | 99.3 | 177 | 98.9 |
| I do not know what they are used for | 4 | 0.8 | 2 | 1.1 |
| **Prior delays in vaccination** | | | | |
| History of prior delays | 72 | 13.4 | 141 | 78.8 |
| No history of prior delays | 464 | 86.6 | 38 | 21.2 |
| **Reasons for prior delays** | | | | |
| Problems with the infant or tutor | 31 | 43.1 | 44 | 31.2 |
| Issues due to the vaccination centers' location, opening hours, accessibility, or distance | 14 | 19.4 | 57 | 40.4 |
| Fear of COVID-19 | 27 | 37.5 | 40 | 28.4 |
| **History of deciding not to vaccinate the infant** | | | | |
| No | 14 | 2.6 | 12 | 6.7 |
| Yes | 522 | 97.4 | 167 | 93.3 |
| **Reasons for not vaccinating the infant** | | | | |
| Fear of vaccine risks | 9 | 64.3 | 8 | 66.7 |
| Fear of COVID-19 | 2 | 14.3 | 3 | 25.0 |
| Expensive complementary vaccines | 3 | 21.4 | 1 | 8.3 |
| **Whether the respondent changed his/her opinion regarding vaccination rejection** | | | | |
| Yes | 7 | 50.0 | 11 | 91.7 |
| No | 7 | 50.0 | 1 | 8.3 |

Table 9: Frequency of responses for each item Trust and Positive Attitudes Towards Vaccines Scale.

|  | Strongly disagree | | Disagree | | Neither agree or disagree | | Agree | | Strongly agree | | Missing  data |
| --- | --- | --- | --- | --- | --- | --- | --- | --- | --- | --- | --- |
| Item | n | % | n | % | n | % | n | % | n | % | n |
| #1 | 2 | 0.3 | 5 | 0.7 | 30 | 4.2 | 188 | 26.3 | 490 | 63.5 | 1 |
| #2 | 2 | 0.3 | 2 | 0.3 | 40 | 5.6 | 110 | 15.4 | 561 | 78.5 | 1 |
| #3 | 0 | 0 | 2 | 0.3 | 37 | 5.2 | 94 | 13.2 | 582 | 81.4 | 1 |
| #4 | 3 | 0.4 | 11 | 1.5 | 63 | 8.8 | 123 | 17.2 | 515 | 72.0 | 1 |
| #5 | 23 | 3.2 | 40 | 5.6 | 73 | 10.2 | 90 | 12.6 | 489 | 68.4 | 1 |
| #6 | 14 | 2.0 | 18 | 2.5 | 72 | 10.1 | 145 | 20.3 | 466 | 65.2 | 1 |
