# Supplementary material for: Delays in the vaccination of infants between 2 and 18 months of age: associated factors in Chile
Source: BMC Public Health. 2023 Sep 28;23:1882. doi: 10.1186/s12889-023-16769-3 (PMC10540413; doi:10.1186/s12889-023-16769-3)
Supplement: Supplementary file 2 — Additional file 2. Annex: Variables include in the questionnaire [file 12889_2023_16769_MOESM2_ESM.docx]

Annex: Variables include in the questionnaire

| **DOMAIN** | **QUESTION** | **ANSWERS** |
| --- | --- | --- |
| 1 | Sex of the tutor | 1= Woman  2= Man |
|  | Relationship between the tutor and the infant | 1= Mother  2= Father  3= Other |
|  | Marital status | 1= Married  2= Single  3= Separated/Divorced/widow |
|  | Family structure | 1= Single parent  2= Biparental  3= Extended family |
|  | Municipality of residence of the tutor | 1= High-income municipality  2= Middle-income municipality  3= Low-income municipality |
|  | Nationality of the tutor and the infant | 1= Chilean  2= Foreigner |
|  | Type of health insurance of the tutor and infant | 1= Public  2= Private |
|  | Number of children of the mother | 1= One child  2= More than one child |
|  | Educational level of the tutor | 1= With no higher education  2= With higher education, unfinished  3= With higher education, finished  4= With postgraduate studies |
|  | Labor condition of the tutor | 1= Unemployed  2= Paid work |
| 2 | Main reason for bringing the infant to this center | 1= To vaccinate the infant  2= Another reason |
|  | The vaccination center location regarding residence | 1= Located in the same municipality of residence  2= Located out of the municipality of residence |
|  | *For those who go to the health center not located in municipality of residence, reasons* | 1= Quality of care  2= Health insurance  3= Closeness  4= Use of health center for other health issues |
|  | Means of transport used to travel to this health care center | 1= Public transport  2= Personal transport |
|  | Time spent traveling to the health care center today | 1= < 30 minutes  2= 30 or more minutes |
|  | Level of satisfaction with several aspects of the vaccination center | Rate the following aspects of the vaccination center with a grade from 1= Worst grade to 7= The best grade.   1. Location 2. Accessibility and distance 3. Opening hours 4. Waiting time 5. Service provided by staff 6. General rating for the vaccination center |
| 3 | Dose scheduled for that day | 1= 2-6 months  2= 18 months |
|  | Has a vaccination card | 1= Yes  2= No |
|  | Knows the date of the next vaccination | 1= Yes  2= No |
|  | *Reasons for ignoring the date of the next vaccination* | 1= I was not told  2= I did not understand, or it was unclear  3= I do not remember  4= Other reasons |
|  | Has been unable to vaccinate the infant in the past | 1= Has been able to do so  2= Has not been able to vaccinate the infant |
|  | *Reasons for being unable to vaccinate the infant* | 1= Vaccination system issues  2= Problems of the infant or tutor |
|  | *And if this has happened more than once* | 1= Yes  2= No |
|  | *Feelings linked to being unable to have the child vaccinated* | 1= It is the way things are for me  2= I became upset  3= I did not mind |
| 4 | I know what vaccines are used for in general | 1= I know what they are used for  2= I do not know what they are used for |
|  | Sources of information accessed | 1= Health professionals  2= Friends  3= Internet |
|  | Prior delays in vaccination | 1= History of prior delays  2= No history of prior delays |
|  | *Reasons for prior delays* | 1= Problems with the infant or tutor  2= Issues due to the vaccination centers´ location, opening hours, accessibility, and/or distance  3= Fear of COVID-19 |
|  | History of deciding not to vaccinate the infant | 1= No  2= Yes |
|  | *Reasons for not vaccinating the infant* | 1= Fear of vaccine risks  2= Expensive complementary vaccines  3= Fear of COVID-19  4= Other reasons |
|  | *Whether the respondent changed his/her opinion regarding vaccination rejection* | 1= Yes  2= No |
| 5. | Trust and Positive Attitudes Towards Vaccines Scale. | What is your degree of agreement with the following statements: 1= Strongly disagree to 5= Strongly agree.   1. Vaccines are good and safe 2. Vaccination protects my child from diseases 3. In general, I do what physicians recommend me regarding the vaccines for my child 4. The information posted in the vaccination calendar seems adequate and I trust it. 5. I am satisfied with all the vaccines that are available in the vaccination schedule. 6. The information I receive about adverse events (or unwanted reactions) seems adequate to me and gives me confidence |
